# Medications Discovery: Importance of Assessment of Drug Self Administration Dose-Effect Curves

**DOI:** 10.4172/2329-6488.1000e121

**Published:** 2015-06-12

**Authors:** Takato Hiranita

**Affiliations:** Division of Neurotoxicology, National Center for Toxicological Research, U.S. Food and Drug Administration, 3900 NCTR Road, Jefferson, AR 72079-9501, USA

There are several procedures for assessment of abuse liability/potential in laboratory animals. Among them is an intravenous (IV) drug self-administration procedure that is the gold standard. For example, the IV drug self-administration procedure has high face, predictive and construct validities [1,2]. In addition, the procedure also has lower rates of false positives and negatives, relative to other procedures [2,3]. For these reasons, the procedure has been employed for assessment of various compounds for abuse potential in humans. For example, drugs abused by humans (cocaine, methamphetamine, heroin, and ketamine) maintain self-administration responding above vehicle levels in rats [4,5]. When such a response is maintained above vehicle levels in laboratory animals, a test compound would likely be reinforcing and have abuse potential in humans.

Likewise, the IV drug self-administration procedure is also useful for assessing various compounds for medications discovery against drug abuse. For example, the opioid antagonist naltrexone can decrease self-administration responding maintained by injections of heroin [6]. When such a response is decreased, the compound “theoretically” should antagonize the reinforcing effects of the self-administered drug and could have a potential for development as an anti-abuse medication. Nonetheless, the overall conclusion is not always correct. For example, decreases in self-administration behavior can be observed with not only antagonism but with a potentiation of the reinforcing effects of the target drug. (Figure 1) shows three representative patterns of shifts in dose-effect curves of drug self-administration. Panel A indicates a leftward shift or a potentiation of the effect. In contrast, panels B and C indicate, respectively, a downward (insurmountable antagonism) and rightward shifts (surmountable antagonism). A leftward shift has been observed when the dopamine uptake inhibitor is pretreated for cocaine self-administration [7], while the dopamine receptor antagonist has been demonstrated to right-shift a dose-effect curve of cocaine self-administration [4]. Further, insurmountable antagonism has been shown when the mu opioid agonist (±)-methadone was pretreated for heroin self-administration [5]. Importantly, decreases in self-administration responding are observed in each descending limb for all three of the patterns. Thus, it is essential to keep in mind that decreases in drug reinforcement could result from antagonism as well as a potentiation.

In summary, it is very important to assess a drug self-administration dose-effect curve including both the ascending and descending limbs, in order to fully assess compounds for anti-abuse medications discovery. Assessment of an entire dose-effect curve might be time-consuming if a single dose per session design is used. However, recent studies indicate the feasibility of a within-session, within-subject design for various drugs of abuse across pharmacological classes [5,8] and across laboratories [9-11].

## Figures and Tables

**Figure 1:**
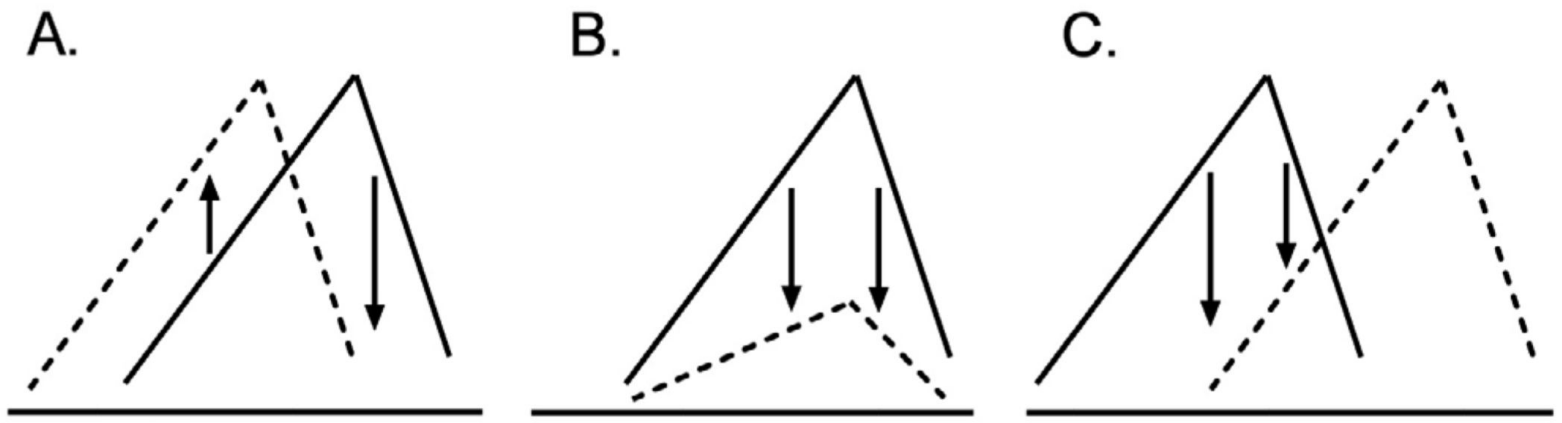
Three representative patterns of shifts in dose-effect curves of drug self administration. Lines indicate basal dose-effect curves. Dashed lines indicate dose-effect curves when pretreated. Panel A. a leftward shift. Panel B. a downward shift. Panel C. a rightward shift.
